# Robert Browning's Views of Life and Death

**Published:** 1890-01-25

**Authors:** 


### Robert Browning's Views of Life and Death.

Though Robert Browning is acknowledged on all sides to be a great poet, to be worthy of our love and admiration, and fit to be honoured by burial in the thrice-hallowed ground of Poet's Corner, yet how many there are who say, wearily, "We cannot understand him," or, "I have tried to read Browning's poetry, and cannot make out the meaning." With these disappointed ones we have considerable sympathy; much of Browning's poetry is confessedly hard reading, but his thoughts are so fine, his hope is so steadfast, his belief in God so undimmed, his faith in humanity and immortality so unquenchable, that we trust that after perusing the following short exposition of his views on life and death many of our readers will seek, not only interest of an intellectual character, but that moral strength and encouragement, which comes from intercourse with a great and noble mind, in the pages of the now dead poet.

Browning never despaired of life; he firmly believed all was working for good. Suffering purged humanity, struggle strengthened, failure chastened, and even sin and crime taught lessons which might never be completely learnt in this darkened and straitened existence, but, hoping still, he declared that

"Things learned on earth we shall practise in heaven."

His philosophy is more fully expressed in the following:

"There shall never be one lost good! What was, shall live as before;
The evil is null, is nought, is silence implying sound;
What was good, shall be good, with, for evil, so much good more;
On earth the broken arcs; in the heaven, a perfect round."

Holding thus the opinion that this life was only a term of probation for another and greater existence, he glorified effort. In the beautiful poem of "Rabbi ben Ezra" this is forcibly expressed:

"Then welcome each rebuff
That turns earth's smoothness rough,
Each sting that bids not sit nor stand, but go!
Be our joy three parts pain!
Strive and hold cheap the strain;
Learn, nor account the pang; dare, never grudge the throe."

It was not, however, success that he valued as the prize of effort. He preached more constantly the view,

"Shall life succeed in that it seems to fail"

and that

"Endeavour's all, success is nought."

but also that it was in aiming at the unrealisable ideal that man was greatest:

"It is not what man does which exalts him, but what man would do."

This is the burden of many of his most beautiful poems and dramas; "Paracelsus," "Luria," "Saul," and "Abt Vogler," for example.

Though a man of great learning and versed in metaphysics and modern science, he never ceased to proclaim, with the insistence and faith of the prophets of old, that God is, and that life is eternal. In one of his earlier poems he says:

"All that is, at all,
Lasts ever, past recall;
Earth changes, but thy soul and God stand sure."

And in one of his later poems he speaks of himself as one who

"At least believed in soul, was very sure of God."

The uses of life are, he taught, simply to learn to love and to know the truth. In "Fifine at the Fair," one of his most delightful poems, in which are freely discussed the uses to which man puts life, and the lessons, often bitter, learnt thereby, he says

"Life means—learning to abhor
The false and love the true, truth treasured snatch by snatch."

Taking life so earnestly, he insisted upon the responsibility of youth, and he says:

"Youth is the only time
To think and decide on a great course,
Manhood with action follows."

And Sordello exclaims:

"Youth once gone is gone;
Deeds let escape are never to be done."

Indeed, not only youth, but all good gifts are, he claims, ours only for the Master's use.

"Where's the use of the lip's red charm,
The heaven of hair, the pride of the brow,
And the blood that blues the inside arm—
Unless we turn as the soul knows how,
The earthly gift to an end divine?"

To one who held so pure a faith with such simplicity and earnestness, it is not surprising that he looked upon death absolutely without alarm and treated it only as a portal to a fuller, freer, and higher life. "Wait death, nor be afraid," he makes Rabbi ben Ezra say, and in that wonderful little gem, "Prospice," in which he begins by asking shall he fear death, then describes its terrors, and finally says:

"I would hate that death bandaged my eyes and forebore,
And bade me creep past.
No, let me taste the whole of it, fare like my peers,
The heroes of old,
Bear the brunt, in a minute pay glad life's arrears
Of pain, darkness, and cold.
For sudden the worst turns the best to the brave,
The black minutes' at end,
And the elements' rage, the fiend voices that rave
Shall dwindle, shall blend,
Shall change, shall become first a peace out of pain,
Then a light, then thy breast,
O, thou soul of my soul! I shall clasp thee again,
And with God be the rest."

This is how he met his death. Death acceded to his wish and did not bandage his eyes. He met "the arch fiend" open-eyed and not afraid; for, as in the last words he published the last day of his life, he was

"One who never turned his back but marched breast forward,

Never doubted clouds would break,

Never dreamed, though right were worsted, wrong would triumph,

Held we fall to rise, are baffled to fight better,

Sleep to wake."

